# Synthesized OVA_323-339_MAP octamers mitigate OVA-induced airway inflammation by regulating Foxp3 T regulatory cells

**DOI:** 10.1186/1471-2172-13-34

**Published:** 2012-07-06

**Authors:** Wen Su, Wenwei Zhong, Yanjie Zhang, Zhenwei Xia

**Affiliations:** 1 Department of Pediatrics, Ruijin Hospital Affiliated to Shanghai Jiao Tong University School of Medicine, Shanghai, China; 2 Department of Pediatrics, Ruijin Hospital, Ruijin 2nd Road 197, Shanghai, 200025, China

**Keywords:** Allergic airway inflammation, Specific immunotherapy, Multiple antigen peptide

## Abstract

**Background:**

Antigen-specific immunotherapy (SIT) has been widely practiced in treating allergic diseases such as asthma. However, this therapy may induce a series of allergic adverse events during treatment. Peptide immunotherapy (PIT) was explored to overcome these disadvantages. We confirmed that multiple antigen peptides (MAPs) do not cause autoimmune responses, which led to the presumption that MAPs intervention could alleviate allergic airway inflammation without inducing adverse effects.

**Results:**

In this study, synthesized OVA_323-339_MAP octamers were subcutaneously injected into ovalbumin (OVA)-sensitized and -challenged Balb/c mice to observe its effect on allergic airway inflammation, Th2 immune response**,** and immune regulating function. It was confirmed that OVA sensitization and challenge led to significant peritracheal inflammatory**,** cell infiltration**,** and intensive Th2 response. Treatment of OVA_323-339_MAP octomers in the airway inflammation mice model increased CD4^+^CD25^+^Foxp3^+^ T regulatory (Treg) cells and their regulatory function in peripheral blood, mediastinal draining lymph nodes, and the spleen. Furthermore, OVA_323-339_MAP increased IL-10 levels in bronchial alveolar lavage fluid (BALF); up-regulated the expression of IL-10, membrane-bound TGF-β1, as well as Foxp3 in lung tissues; and up-regulated programmed death-1 (PD-1) and cytotoxic T lymphocyte associated antigen 4 (CTLA-4) on the surface of Treg cells. These results were further correlated with the decreased OVA specific immunoglobulin E (sIgE) level and the infiltration of inflammatory cells such as eosinophils and lymphocytes in BALF. However, OVA_323-339_ peptide monomers did not show any of the mentioned effects in the same animal model.

**Conclusions:**

Our study indicates that OVA_323-339_MAP had significant therapeutic effects on mice allergic airway inflammation by regulating the balance of Th1/Th2 response through Treg cells in vivo*.*

## Background

Bronchial asthma, one of the most common chronic inflammatory diseases, has complex pathogenic mechanisms. As an allergic disease, it is mediated mainly by Th2 immune responses, and is affected by genetic as well as environmental factors. Many approaches for treating this disease are palliative rather than disease modifying. In the past century, antigen-specific immunotherapy (SIT) has been widely practiced in treating allergic diseases, and has been a form of disease-modifying treatment, which has been demonstrated to be clinically efficacious in asthma [1,2]. However, the SIT desensitizers commonly used presently are whole allergen preparations that have the potential to induce a series of adverse allergic events that can, at times be fatal [3]. On this basis, many approaches that can reduce the allergenicity of immunotherapy preparations and maintain their immunogenicity are under development. One of these approaches is peptide immunotherapy (PIT), which utilizes synthesized short peptides containing major T cell epitopes of the allergen. This approach could present allergen-derived T cell epitopes while avoiding the immunoglobulin E (IgE) mediated mast cells or basophils. Synthesized short peptides containing major T cell epitopes of cat allergen Fel d1, dust mite allergen Der p2, birch pollen allergen Bet v1 and bee venom allergen Api m1 have been demonstrated to be efficacious in experimental animal models in recent years [4-7].

Clinical studies have also shown that in patients allergic to cats, rhinitis symptom scores, asthma symptom scores, and lung function were all improved markedly after peptide treatment [8-14]. However, some conflicting results were also reported. Janssen et al. [15] found that OVA_323-339_ peptides containing major T cell epitopes of ovalbumin (OVA) proteins do not mitigate the effects of airway inflammation, but conversely, aggravate disease in OVA-induced asthmatic mice. The same result has been observed in encephalomyelitis (EAE) mice model. Wegman et al.[16] found that PLP_139-151_ peptide (proteolipid protein) monomers had no effect in improving disease, while synthesized PLP_139-151_ multiple antigen peptide (MAP) octamers successfully inhibited the occurrence of EAE induced by encephalitis pathogenic protein, thus demonstrating that peptide monomers processing can alter their immunological characteristics.

MAPs are dendriform peptides, which can be tetramers or more typically, octamers. Each peptide monomer is independently and covalently linked to a branched central lysine matrix. MAPs can induce high levels of immune response and have been used in vaccine development for a variety of infectious diseases [17,18]. In addition, MAPs can also enhance the peptide-specific T cell response and play a role in the protective immune response [19-22]. Also, Wegman et al.[16] further expanded the scope of MAPs application in studies of autoimmune diseases. There have been no reports of studies concerning the treatment of allergic airway inflammation using MAPs. Therefore, in the present study, eight OVA_323-339_ short peptides were integrated on a lysine core matrix to constitute OVA_323-339_MAP octamers, which were then subcutaneously injected into OVA-induced allergic airway inflammation mice. The inhibitory effect of OVA_323-339_MAP on airway inflammation and Th2 immune response were observed and compared with OVA_323-339_ monomers.

Based on the current theories, T regulatory (Treg) cells proliferation is crucial in maintaining homeostasis. However, existing research on the role of Treg cells in PIT treatment remain controversial [23,24]. In our preliminary study, we found that Treg cells played an important immunoregulatory role in the development and progression of airway inflammation in mice [25,26]. Thus, the changes of Treg cells after OVA_323-339_ MAP octamer treatment were further explored in this study.

## Results

### OVA_323-339_MAP intervention attenuates OVA-induced airway inflammation

**Figure 1:**
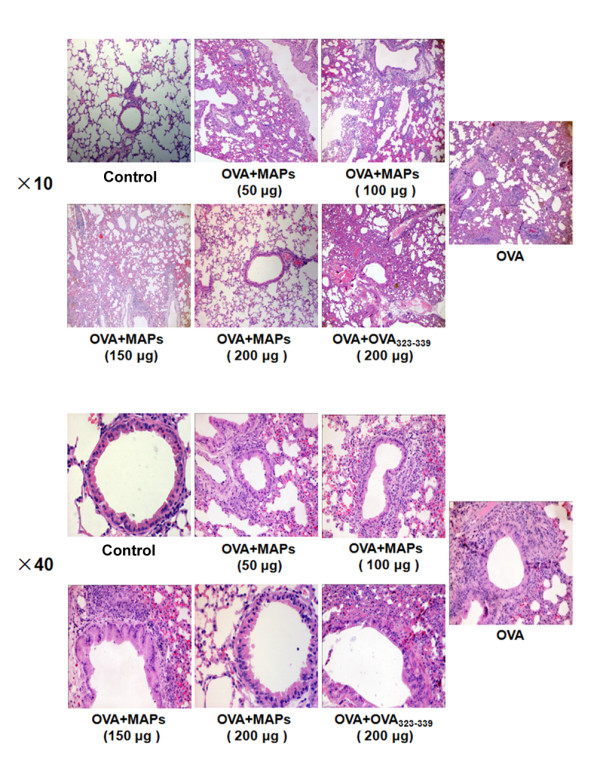
**Impact of OVA**_**323-339**_**and OVA**_**323-339**_**MAP intervention on airway inflammation in mice.** After OVA sensitization and challenge, typical airway inflammation was observed in Balb/c mice. The airway inflammation was not significantly alleviated by subcutaneous injection of 200 μg of OVA_323-339_ peptide monomers. However, OVA_323-339_MAP, containing the equivalent weight of peptides, significantly reduced peritracheal inflammatory cells infiltration and this effect was dose-dependent. Data represent one of three independent experiments.

**Figure 2:**
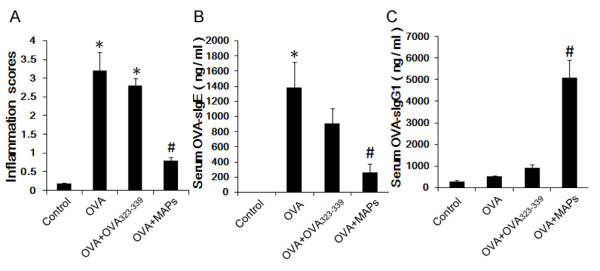
**Peribronchila inflammation score and immunoglobulin levels in serum.** The peribronchial inflammation score and OVA-sIgE levels in OVA group and OVA_323-339_ group were significantly increased, but they were significantly decreased accompanied by increased OVA-sIgG1 after OVA_323-339_MAP treatment (* compared with the control group, *P* < 0.05; # compared with the OVA_323-339_ group, *P* < 0.05). Data represent one of three independent experiments.

Recently, MAPs have been shown to alleviate the severity and block the progress of EAE [16]. This finding emphasizes the potential of MAPs intervention as an effective immunotherapy to treat antigen-specific allergic diseases. Thus, to examine the impact of MAPs on allergic lung disease, we sensitized Balb/c mice with OVA emulsified in Al(OH)_3_ and induced airway inflammation by intranasal administration of the antigen. As illustrated in Figures 1 and 2 A, OVA priming and activation led to a high inflammation score associated with marked peribronchial leukocyte infiltration, edema, and epithelial damage. Systemic administration of regular OVA_323-339_ peptide before local antigen activation did not alter the severity of the airway inflammation and tissue injury, whereas treatment with OVA_323-339_MAP substantially reduced the inflammatory response in a dose-dependent manner. In addition to the histological evaluation, we assessed airway inflammation by examining total cell counts and eosinophil, neutrophil, lymphocytes and macrophages counts in bronchial alveolar lavage fluid (BALF) of these mice. Compared to the normal control group, OVA sensitization and challenge induced a significant increase of total cells, eosinophils, neutrophils, lymphocytes and macrophages counts, OVA_323-339_ peptide monomers intervention mildly reduced the total cell count and EOS number. However, a significant decrease in total cell as well as EOS and LYM infiltrates was observed in OVA_323-339_MAP treated mice (Table 1).

**Table 1:** **Total Cells and Differential Cells Counts in BALF (×10**^**4**^**)**

	**Total cells**	**Eosinophils**	**Neutrophils**	**Lymphocytes**	**Macrophages**
Control	8.91 ± 1.17	0.00 ± 0.00	0.05 ± 0.00	0.19 ± 0.01	8.37 ± 1.57
OVA	74.64 ± 12.60 *	9.08 ± 2.20 *	2.12 ± 0.52 *	4.51 ± 1.43 *	58.41 ± 10.05 *
OVA + OVA_323-339_	63.64 ± 8.07 #	4.58 ± 0.86 #	1.03 ± 0.15	3.06 ± 0.52	55.59 ± 9.91
OVA + MAPs	43.32 ± 5.64 §	1.02 ± 0.16 §	0.92 ± 0.16	0.86 ± 0.11 §	40.24 ± 6.29

*P < 0.05 Compared with Control group.

#*P* < 0.05 Compared with OVA group.

§*P* < 0.05 Compared with OVA + OVA_323-339_ group.

Antigen specific immunoglobulin E (sIgE) plays an important role in allergic cell degranulation and hyperresponsiveness. Therefore, we tested whether MAPs intervention altered immunoglobulin production and switching. As shown in Figure 2 B, no OVA-sIgE was detected in the serum of control animals, whereas OVA challenge led to a robust production of IgE that specifically recognizes OVA. Conversely, OVA_323-339_MAP treatment significantly suppressed the level of OVA-sIgE. Interestingly, the mice administered with OVA_323-339_MAP had a substantial elevation of OVA-specific immunoglobulin G_1_ (sIgG_1_) which is the indicator for the success of immunotherapy, compared to the control and OVA_323-339_ peptide monomers treated groups (Figure 2 C). This data suggest that OVA_323-339_MAP intervention may mitigate the antigen-specific allergic process and response.

Based on the above results, we further explored how the OVA_323-339_MAP mitigates the airway allergic airway.

### OVA_323-339_MAP intervention alters the OVA-induced Th2 response

**Figure 3:**
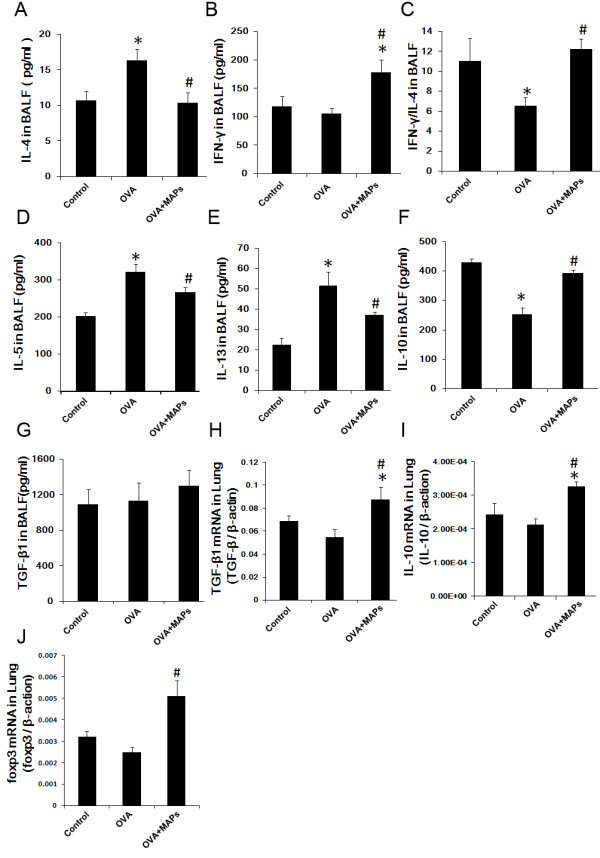
**The levels of cytokines in BALF and in the lung.** OVA_323-339_MAP treatment could reverse OVA-induced response, reducing the IL-4, IL-5**,** and IL-13 levels, increasing the level of IFN-γ and IL-10 and regulating the ratio of IFN-γ/IL-4. For the level of TGF-β1 in BALF, there were no markedly differences in each group, but the TGF-β1 mRNA expression was enhanced significantly after OVA_323-339_MAP intervention. In addition, the expression levels of IL-10 and Foxp3 in the lung tissue were higher in MAPs group than that of control and OVA groups. (* compared with the control group, *P* < 0.05; # with the OVA group, *P* < 0.05). Data represent one of three independent experiments.

**Figure 4:**
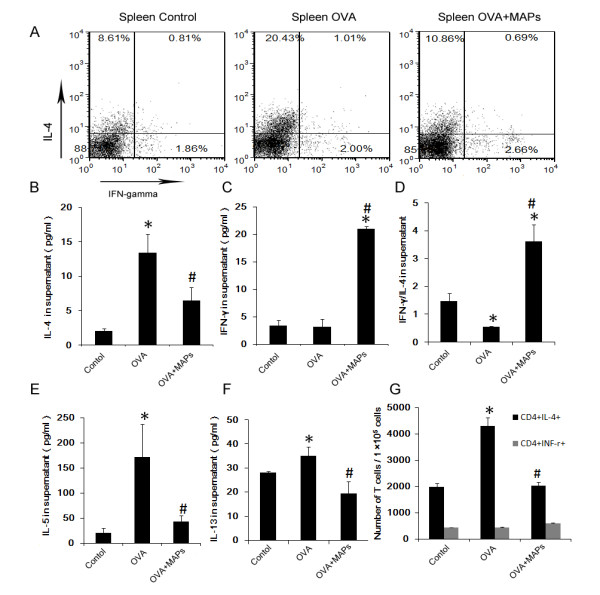
**Th2 cells differentiation in spleen.** Splenocytes were co-cultured with OVA (1 mg/ml) in vitro for the examination of Th2 cells differentiation. OVA_323-339_MAP intervention could significantly reduce the ratio of CD4^+^IL-4^+^ T cells compared with the OVA group (gating on CD4^+^ T cell populations). ELISA detection of the cytokines in supernatant showed that IL-4, IL-5, and IL-13 levels were significantly increased in the OVA group compared with the control group, but OVA_323-339_MAP intervention could significantly suppress their secretion and enhance the expression of IFN-γ, then the ratio of IFN-γ/IL-4 was increased. (* compared with the control group, *P* < 0.05; # compared with the OVA group, *P* < 0.05). Data represent one of three independent experiments.

It is well documented that OVA-induced airway inflammation in Balb/c mice is mainly mediated by a Th2 response. In light of the finding that OVA_323-339_MAP can attenuate allergic airway inflammation, we asked if OVA_323-339_MAP treatment alters the OVA-induced Th2 response in the host. To this end, we measured the expression of Th1/Th2-related cytokines (IFN-γ, IL-4, IL-5 and IL-13) and the ratio of IFN-γ/IL-4 in the BALF of these mice. The levels of IL-4, IL-5 and IL-13 were significantly elevated in mice challenged with OVA (Figure 3 A, D, E), while the ratio of IFN-γ/IL-4 decreased significantly (Figure 3 C). However, OVA_323-339_MAP intervention reduced local Th2 cytokines production and increased the level of IFN-γ and the ratio of IFN-γ/IL-4 (Figure 3 A ~ E). Furthermore, in order to determine if OVA_323-339_MAP treatment influences antigen recall response in mice sensitized with OVA, splenocytes from control and OVA-challenged mice with or without OVA_323-339_MAP intervention were harvested. The IL-4 and IFN-γ positive populations were analyzed by flow cytometry (FCM) in CD4^+^ T cells and the amount of IL-4, IL-5, IL-13 and IFN-γ in the cell culture media were quantified by ELISA. When the lymphocytes were re-stimulated with OVA in vitro, CD4^+^ T cells from OVA-challenged mice exhibited a significantly higher IL-4 production than the control group. In vivo treatment with OVA_323-339_MAP virtually blocked Th2 cytokines production in response to OVA re-stimulation (Figure 4 A, G). Likewise, ELISA demonstrated that splenocytes from OVA-challenged mice exhibited a significantly higher IL-4, IL-5 and IL-13 response to antigen re-stimulation, while this antigen recall response was diminished in OVA_323-339_MAP-treated mice (Figure 4 B, E, F). Interestingly, OVA_323-339_MAP intervention caused more IFN-γ products while the levels of IFN-γ between the control and airway inflammation groups were biologically insignificant (Figure 4 C). These results suggest that OVA_323-339_MAP may alter Th2 response in this allergic airway model.

OVA_323-339_MAP intervention increased the populations of CD4^+^CD25^+^Foxp3^+^ Treg cells, up-regulated the production of IL-10, membrane-bound TGF-β1 and Foxp3 in the lung, as well as the surface expression of PD-1 and CTLA-4

**Figure 5:**
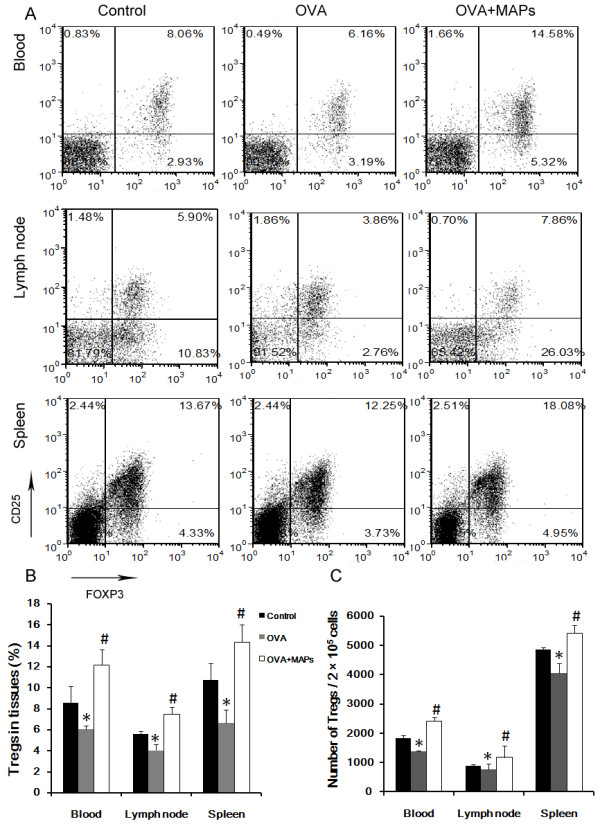
**FCM detection of CD4**^**+**^**CD25**^**+**^**Foxp3**^**+**^**Treg cells in peripheral blood, mediastinal draining lymph nodes, and spleen of mice.** In vivo OVA challenge could decrease the level of Treg cells in the mice. However, the levels of local and peripheral CD4^+^CD25^+^Foxp3^+^ Treg cells was about 0.5-1.0-fold increase after OVA_323-339_MAP treatment (gating on CD4^+^ T cell populations, * compared with the control group, *P* < 0.05; # compared with the OVA group, *P* < 0.05). Data represent one of three independent experiments.

In light of these findings, we evaluated whether OVA_323-339_MAP induces peripheral tolerance as a part of a potential mechanism for mitigating OVA-induced airway inflammation. Treg cells regulate the functions of other CD4^+^CD25^-^ effector cells and play an important role in balancing Th1/Th2 cell differentiation. Therefore, we observed the alteration of Treg cells. CD4^+^CD25^+^Foxp3^+^ Treg cells in peripheral blood, mediastinal draining lymph nodes, and spleen were measured by FCM. In vivo OVA challenge decreased the level of Treg cells in the mice. However, OVA_323-339_MAP increased the population of local and peripheral CD4^+^CD25^+^Foxp3^+^ Treg cells approximately 0.5-1.0 fold (Figure 5). In addition, we further detected the Foxp3 transcription expression in the lung to observed Treg cells in inflamed local tissue. The result showed that Foxp3 expression in the lung was augmented by MAPs intervention (Figure 3 J).

CD4^+^CD25^+^Foxp3^+^ Treg cells exert their regulatory effect on immune effector cells by both direct contact and indirect suppression. The indirect suppression is mediated by anti-inflammatory cytokines including IL-10 and transforming growth factor (TGF)-β1. Thus, we measured IL-10 and TGF-β1 levels in BALF and their expression in lung tissue. As shown in Figure 3 F and I, OVA challenge significantly decreased the level of IL-10 in BALF and lung tissue, but OVA_323-339_MAP intervention increased its concentration. Although the level of TGF-β1 in BALF was not affected by MAPs (Figure 3 G), the transcription of TGF-β1 in lung was significantly decreased in OVA challenge mice when compared to control mice. However, it is important to note that OVA_323-339_MAP intervention could reverse this change which is consistent with the results shown in our previous publication (Figure 3 H) [25].

**Figure 6:**
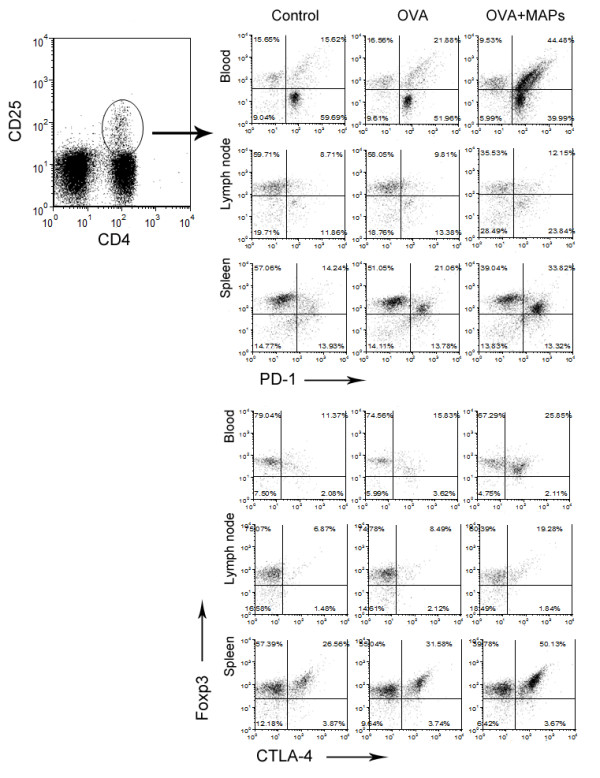
**FCM detection of PD-1 and CTLA-4 on the surface of CD4**^**+**^**CD25**^**+**^**Foxp3**^**+**^**Treg cells in peripheral blood, mediastinal draining lymph nodes, and spleen of mice.** To evaluate whether Treg cells in this study could play their role by contact inhibition, the surface expression of two major negative co-stimulatory molecules were detected by FCM. Freshly isolated cells from blood, mediastinal draining lymph nodes and spleen were stained with fluorescently antibodies for CD4, CD25, Foxp3 and PD-1 or CTLA-4. The results showed a significantly higher levels of PD-1 and CTLA-4 on the surface of Treg cells in MAPs treated mice than in control mice and OVA treated mice (gating on CD4^+^CD25^+^ cells).

Cell-cell contacting is the other mechanism of Treg function, co-stimulatory molecules such as programmed death (PD)-1 and cytotoxic T lymphocyte associated antigen (CTLA)-4 contribute to the regulatory function of Treg cells. Therefore, we investigated the expression of PD-1 and CTLA-4 on the surface of Treg cells by FCM. Interestingly, compared to the control mice, OVA challenge elevated the expression of PD-1 mildly, however, the ratio of PD-1^+^ Treg cells increased significantly after treatment with OVA_323-339_MAP in blood, mediastinal draining lymph nodes, and spleen. The similar alteration of CTLA-4 expression on the surface of Treg cells was observed in same tissues (Figure 6).

**Figure 7:**
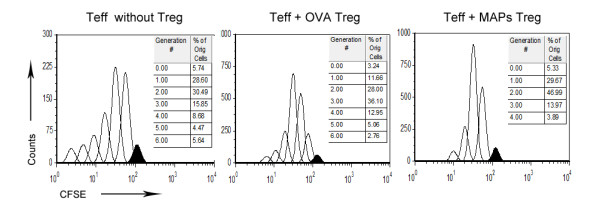
**FCM detection of the inhibitory effect of Treg cells on Teff proliferation.** CD4^+^CD25^+^ T cells from normal mice and OVA-challenged with or without intervention mice were isolated using immunomagnetic beads, then co-cultured with effector CD4^+^ lymphocytes and CD11c^+^ APC from normal mice for 5 days with OVA (1 mg/ml) stimulation. Effector T cells proliferation in response to OVA stimulation was assessed by FCM. The results showed that the proliferation cycles of Teff cells co-cultured with OVA_323-339_MAP group were four cycles, less than that in the OVA group and Teff without Treg cells group (six cycles). Data represent one of three independent experiments.

To further assess the function of Treg cells in these mice, we isolated CD4^+^CD25^+^ Treg cells from OVA-challenged mice, with or without OVA_323-339_MAP treatment. The Treg cells were co-cultured with effector CD4^+^ lymphocytes and CD11c^+^ antigen presenting cells (APCs) from the normal untreated mice. Five days later, effector T cells proliferation in response to in vitro OVA stimulation was assessed by FCM. The results showed that effector T cells underwent six proliferation cycles when they were cultured with Treg cells from OVA-challenged mice, the same as that of effector T cells without Treg. Despite OVA challenge, the Treg cells from the mice treated with OVA_323-339_MAP displayed a regulatory function, as evidenced by reduced effector T cell proliferation cycles (Figure 7).

## Discussion

The OVA_323-339_ peptides used in this experiment contained major T cell epitopes, representing major peptides that could be presented to Th cells through MHC class II molecules after APCs processing of OVA proteins. Structural analysis has confirmed that OVA_323-339_ peptides contain multiple loci that can be combined with MHC class II molecules and TCRs to form an immune sandwich complex and induce an immune response [27]. It has also been demonstrated that after OVA_323-339_ peptide challenge, 82% of OVA-sensitized mice developed immediate hypersensitivity. In addition, OVA and OVA_323-339_ peptides are equivalent in increasing mice airway reaction during challenge test [28]. Thus, OVA_323-339_ peptides have been considered for use in PIT in OVA-induced asthma in mice. However, Janssen et al.[15] found that after using these peptides for immunotherapy in mice, neither airway inflammation was alleviated nor were OVA-sIgE levels and Th2 immune responses suppressed, which are the opposite of the expected results. In our study, similar results were found using OVA_323-339_ peptide monomers, while after OVA_323-339_MAP intervention, airway inflammation in mice was significantly alleviated and EOS infiltration decreased markedly. These results suggest that OVA_323-339_MAP, instead of OVA_323-339_ peptide monomers have a significant antagonizing effect on airway inflammation. The previous reports confirmed that the branching lysine core is an immunologically silent dendrimeric structure [29], therefore, the effects of MAPs were not caused by the lysine core.

In addition, the effect of OVA_323-339_MAP on airway inflammation was dose-dependent. In clinical study, it has been suggested that a high-dose antigen can induce T cell unresponsiveness and also trigger apoptosis of Th2 cells in allergen stimulated periphery blood mononuclear cell (PBMC) from patients receiving SIT treatement [30-33]. Further analysis found that after MAPs intervention, the level of OVA-sIgE in periphery blood was significantly reduced, while OVA-sIgG_1_ level was significantly increased. These results indicated that OVA_323-339_MAP intervention could affect the class-switch of OVA-specific immunoglobulin. Clinical trials have shown that allergen-sIgE level gradually decrease in allergic patients as a successful treatment proceeds, while the levels of sIgGs, especially IgG_1_ and IgG_4_ gradually increase [34,35]. Therefore, immunoglobulin class-switch is considered to be one of the important indicators reflecting the success of immunotherapy [36,37]. These results also showed that OVA_323-339_MAP could alter the immunological characteristics of monomer peptides after polymerization and possessed the opposite effect to peptide monomers. Wegman’s study[16] also supported this conclusion. They found that PLP_139-151_MAP instead of PLP_139-151_ peptide monomer applied for intervention in encephalitogenic proteolipid protein PLP_139-151_ peptide-induced EAE mice could prevent antigen-specific T cells trafficking into the brain, thereby inhibiting inflammation. Our study showed that OVA_323-339_MAP suppressed Th2 immune response; reduced IL-4, IL-5, and IL-13 levels in either BALF or splenocyte culture supernatant significantly as well as the ratio of Th2 cells in spleen concurrently. Up-regulated ratio of IFN-γ/IL-4 reflected that MAPs could affect the Th1/Th2 immune balance by inhibiting Th2 cell differentiation and Th2 immune responses.

It is well documented that Treg cells are essential for regulating immune effector cells, balancing Th1/Th2 response. They are a marker of tolerance induction in controlling autoimmune diseases and maintaining immune tolerance. CD4^+^CD25^+^ cells are a well characterized Treg population and possess potent immunosuppressive functions. Thus, we examined the quantity of Treg cells in our model. The results showed that OVA_323-339_MAP intervention significantly increased the ratio of CD4^+^CD25^+^Foxp3^+^ Treg cells in peripheral blood, mediastinal draining lymph nodes, and spleen of mice. Transcription factor Foxp3 is not only highly expressed in Treg cells, but also correlated with Treg activation. Thus, we further detected the level of transcription factor Foxp3 in the lung tissue by real-time PCR to evaluate the effect of Treg cells in lung tissue. The results indicated that the expression of Foxp3 is augmented significantly in MAPs treated mice. IL-10 is an anti-inflammatory cytokine produced by Treg cells. CD4^+^CD25^+^Foxp3^+^ Treg cells exert their anti-inflammatory effect through IL-10, which also promotes the conversion of CD4^+^CD25^-^ Treg cells to CD4^+^CD25^+^ Treg cells. IL-10 suppresses T cells by blocking CD2, CD28, and inducible costimulatory molecule (ICOS) signals through a rapid signal transduction cascade, leading to T cell tolerance. IL-10 can also down-regulate the expression of MHC class II molecules and costimulatory molecules of monocytes and DCs, thus inhibiting the antigen-presenting function of these cells. In addition, IL-10 could also regulate the humoral immune response by promoting class-switch of immunoglobulin and up-regulating IgG production [38,39]. In the patients receiving PIT of bee venom or cat allergen, the IL-10 levels significantly increased [13,40]. For this reason, we further observed the changes of IL-10 levels in different groups. Our study showed that OVA_323-339_MAP could increase the IL-10 level in OVA challenge mice to a normal level, indicating that inducing IL-10 secretion may be one of the functions of OVA_323-339_MAP. Besides its tolerance-inducing capability, peptide immunotherapy-induced IL-10-dependent immune tolerance correlates to inhibition of the “linkage epitope” response [41]. In our study, subcutaneous injection of OVA_323-339_MAP could alleviate airway inflammation development and Th2 immune response in mice challenged by the OVA protein, suggesting that OVA_323-339_MAP therapy could inhibit in vivo immune response to the entire OVA protein.

It is acknowledged that TGF-β1 could induce the conversion of naïve CD4^+^CD25^-^ T cells into CD4^+^CD25^+^ T cells by up-regulating Foxp3 expression, and play an important role in enhancing the immunosuppressive capacity of CD4^+^CD25^+^ T cells [42,43]. Interestingly, the levels of TGF-β1 in BALF of each group did not have marked differences, while TGF-β1 transcription level in the lung of OVA_323-339_MAP group was increased when compared with the OVA group. The same phenomenon had been observed in our previous study, suggesting that membrane-bound TGF-β1 in CD4^+^CD25^+^ Treg cells was elevated [25]. Some reports have demonstrated that Treg cells could induce immune tolerance by cell-cell contacting directly with membrane-bound TGF-β1 being essential for this regulation, and it also could mitigate allergic airway inflammation through a Notch1-mediated mechanism [44,45]. It has been reported that PD-1 and CTLA-4 contribute to the suppressive activity of allergen-specific regulatory cells, and are involved in the peripheral tolerance. Several studies have confirmed that PD-1 and its ligands (PD-L1 and PD-L2) as well as CTLA-4 and ligands (B7-1 and B7-2) could drive the differentiation of CD4^+^Foxp3^+^ T cells, down-regulate contact hypersensitivity reaction, reduce airway hyperreactivity, prevent eosinophil infiltration in the lungs, and prevent IgE production [46-51]. Piconi et al. also found that during allergen-specific immunotherapy, the expression of PD-L1 is increased compared with untreated control group [52], which indirectly indicated that PD-1 played an important role in inducing immune tolerance. In our study, we found that the expression of PD-1 and CTLA-4 on Treg cells in blood, mediastinal draining lymph nodes, and spleen did not alter noticeably between control and OVA groups, whereas OVA_323-339_MAP intervention significantly increased the expression of two molecules on the surface of Treg cells. It also indicated that OVA_323-339_MAP induced immune tolerance through cell-cell contacting suppression is mediated by membrane-bound TGF-β1, as well as PD-1 and CTLA-4.

As both cytokines and costimulatory molecules are key factors for Treg cells activity, we investigated the suppressive function of Treg cells. The function detection also showed that the inhibitory effect of Treg cell from the OVA_323-339_MAP intervention mice on effector T cells (Teff) proliferation was superior to that of OVA challenged mice. However, in clinical studies of PIT, there is still controversy over the role of Treg cells. After treatment with major T cell epitope of cat allergen Fel d1, the Th2 immune response was inhibited in vivo, but the inhibitory function of CD4^+^CD25^+^ Treg cells was not significantly enhanced [24]. In another study on Fel d1, after peptide treatment, a group of CD4^+^ T cells with a regulatory effect significantly increased and could inhibit CD4^-^ T cells function [23]. In addition, Wegman et al.[16] found that MAPs treatment did not have a clear effect on CD4^+^Foxp3^+^ cells in the mouse spleen and central nervous system. However, our research showed that OVA_323-339_MAP intervention could not only increase the quantity of Treg cells, but also enhance their function, accompanied by inversing Th1/Th2 balance, regulating humoral immunity, and alleviating mouse airway inflammation.

## Conclusions

This study demonstrated that OVA_323-339_MAP, being different from OVA_323-339_ peptide, could inhibit the Th2 immune response and alleviate allergic airway inflammation in mice model and this function may be mediated by Treg cells through direct and indirect mechanisms.

## Methods

### Experimental materials

In all experiments, 6 to 8 week-old female Balb/c mice (purchased from the Shanghai SLAC Laboratory Animal Co., Ltd.) were allocated randomly to either the experimental group or the control group after 1-week adaptive feeding under SPF conditions (n = 6 in each group). The animal experiment protocols were approved by the Ethics Committee of Ruijin Hospital Affiliated to Shanghai Jiao Tong University School of Medicine. OVA_323-339_MAP octamers were kindly provided by Dr. Dave J Hinrichs at Portland VA Medical Center. OVA_323-339_MAP is similar to PLP_139-151_MAP as described by Wegman and Hinrichs [16], The MAP core is almost the same and the only difference between them is the sequence of the leading peptide. The amino acid sequence of the OVA_323-339_ peptide was Ile-Ser-Gln-Ala-Val-His-Ala-Ala-His-Ala-Glu-Ile-Asn-Glu-Ala-Gly-Arg.

### Preparation of the animal model and intervention therapy

Airway inflammation mouse model was constructed and the mice were interfered according to previous reports [15,25,53]. In brief, each mouse involved with airway inflammation (OVA group) received an intraperitoneal injection of 100 μg OVA (V grade, Sigma, USA) in 0.2 ml Al(OH)_3_ adjuvant suspension on day 0 and day 14, respectively. Following the OVA injection on day 14, the mice were immediately intranasal challenged with 100 μg of OVA in 50 μl saline after anaesthetization via inhalation of isoflurane. The same treatment was conducted from day 32 to day 39, respectively. In the control group, mice received the same volume of Al(OH)_3_ to sensitize and saline to challenge. The intervention therapy was started on day 27 and repeated on day 30. In OVA_323-339_ group, 200 μg of OVA_323-339_ peptide monomers (Anaspec, USA) in 200 μl saline was injected subcutaneously into the back of mice. In the OVA_323-339_MAP group, a series concentration of OVA_323-339_MAP octamers (57.2, 114.4, 171.8 and 228.8 μg of OVA_323-339_MAP equals to 50, 100, 150 and 200 μg of OVA_323-339_ peptides, respectively) was injected in the same way. The percentage of the lysine core matrix in total mass was about 12.6% [16]. The concentration series of OVA_323-339_MAP octamers were administrated to evaluate the dose-effects of OVA_323-339_MAP in airway inflammation mice. For comparison, on the same day of treatment, equivalent volume of saline instead of the OVA_323-339_ peptide monomers or OVA_323-339_MAP octamers was subcutaneously injected in the control and OVA groups.

### Tissue harvest

Twenty-four hour after the final challenge (day 40), all mice were anaesthetized with isoflurane and sacrificed after collecting blood via ophthalmic vein. For each mouse, the trachea was exposed by blunt dissection. Pre-cooled saline (0.4 ml) was slowly injected into the trachea using a 22-gauge i.v. catheter for 3 times, the BALF was collected. This procedure recovered 80 to 90% of the infused fluid.

After collecting BALF, mediastinal lymph nodes, spleens**,** and lungs were collected. The right inferior lung was fixed in 10% neutral formalin for 48 hours, embedded in paraffin, and sectioned (4 μm) for hematoxylin-eosin (HE) staining to observe airway inflammation under the microscope (Olympus AX70, Japan). The left lung was also removed and homogenized for RNA extraction.

### Cell counts in BALF and analysis of peribronchial inflammation

The collected BALF was centrifuged at 453 g at 4°C for 5 minutes. The supernatant was then stored at -80°C before further study. The cell pellet was resuspended in 500 μl saline and counted using a blood cell counting plate. A small amount of the cell suspension was fixed in 95% ethanol for 30 minutes for routine HE staining. A total of 200 cells were randomly selected to calculate EOS/NEU/MAC/LYM and their proportion under the microscope (Olympus AX70, Japan). Different cell counts were calculated by the following equation:

(1) $$\text{Total number}=\left(\frac{\text{number of target cell under the microscope}}{200}\right)\times \text{total cell count}$$

The severity of peribronchial inflammation was graded semiquantitatively according **to** the published reference [54] for the following features: 0: normal; 1: few cells; 2: a ring of inflammatory cells, 1 cell layer deep; 3: a ring of inflammatory cells, 2–4 cells deep; 4: a ring of inflammatory cells of 4 cells deep.

### Cell culture

The single cell suspensions from the mediastinal lymph nodes and spleens were prepared under sterile conditions. Then RPMI-1640 complete culture medium (containing 10% fetal bovine serum, 1% penicillin/streptomycin, 0.1% 2-mercaptoethanol, Gibco, USA) was added to adjust cell concentration to 5 × 10^6^/ml and these cells were seeded into a 24-well cell culture plate (Corning, USA). The cells were then stimulated with 1 mg/ml OVA and incubated in the CO_2_ incubator with 5% CO_2_ and 95% humidity at 37°C. Five days later, all wells were replaced with 1 ml fresh RPMI-1640 complete culture medium without OVA, and cultivated overnight under the same culture condition. Six hours before cells collection, 50 ng/ml phorbol myristoyl acetate (PMA, Sigma, USA), 1 μg/ml ionomycin (Sigma, USA) and 1:1000 Brefeldin A (BFA, eBioscience, USA) were added into each well to stimulate the cytokines secretion. The primary culture supernatant was obtained for cytokines detection and the cells were collected and rinsed in PBS for FCM analysis.

### ELISA

For the detection of OVA-sIgE, a 96-well ELISA plate (Corning, USA) was coated with 100 μl coating buffer (0.1 mol/L carbonate buffer, pH 9.5) containing OVA (10 μg/ml), and then sealed and stored at 4°C overnight. The plate was then washed three times with Phosphate Buffered Saline (PBS) containing 0.05% Tween20 (PBS/T), blocked with 200 μl blocking buffer (PBS containing 10% BSA), and incubated for 2 hours. After washing the plate, 100 μl of each serum sample (diluted at 1:100) or mouse OVA sIgE standards (Serotec, UK) were added in the well. The standard was diluted into a final concentration series of 10000, 5000, 2500, 1250, 625, 312.5, 156.3 and 19.5 ng/ml, respectively. The plate was incubated at 37°C for 2 hours. After rinsing with PBS, 100 μl of horseradish peroxidase (HRP)-goat anti-mouse IgE antibody (Serotec, UK, 1:5000) were added into each well, and incubated at 37°C for additional 2 hours and rinsed for detection.

For the detection of OVA-sIgG, a 96-well ELISA plate was coated with 100 μl of coating buffer containing rabbit anti-mouse IgG (H + L) (SouthernBiotech, USA, 1:8000) for each standard curve well and 100 μl coating buffer containing OVA (1 μg/ml) for each sample well. The plate was incubated at 4°C overnight, washed with PBS/T three times, and then blocked for 2 hours at room temperature. After rinsing, 100 μl mouse IgG1 standards (Southern Biotech, USA) of the dilution concentrations 1000, 200, 40, 8, 1.6, 0.32 and 0.16 ng/ml were added to the standard curve wells and 100 μl serum sample (1:500) were added into the OVA coated wells. The plate was incubated at room temperature for 2 hours and rinsed with PBS/T. Finally, 100 μl of HRP-goat anti-mouse IgG1 antibody (1:32000, SouthernBioetch, USA) were added and incubated at room temperature for 2 hours and rinsed for analysis.

For detection, 100 μl of tetramethyl benzidine dihydrochloride (TMB) chromogenic reagent was added to each well, and the plate was incubated at room temperature for 15 minutes before 100 μl H_2_SO_4_ (1 mol/L) was added to terminate the reactions. The absorbance was determined at 450 nm wavelength and the concentration of OVA-sIgE or OVA-sIgG of each sample was calculated based on the standard curve.

The levels of IL-4, IFN-γ, IL-5, IL-13, IL-10**,** and TGF-β1 in BALF or cell culture supernatant were determined using ELISA kits according to the manufacturer’s instructions (Biolegend, USA or eBioscience, USA).

### Real-time PCR

After collecting BALF, the mice left lung was removed and homogenized, total RNA was extracted by Trizol (Invitrogen Life Technologies). cDNA samples were obtained by PrimeScript reverse transcriptase (Takara, Japan). Real-time PCR was performed using an ABI Prism 7900HT (Applied Biosysterms, Foster Ctiy, CA) according to the manufacturer’s instructions. Primer sequences specific for β-actin is forward primer 5’-CTA AGG CCA ACC GTG AAA AG-3’ and reverse primer 5’-AGC CTG GAT GGC TAC GTA CAT-3’. Primer sequences specific for IL-10 is forward primer 5’-AGA AGC ATG GCC CAG AAA TCA-3’ and reverse primer 5’-GGC CTT GTA GAC ACC TTG GT-3’. Primer sequences specific for TGF-β1 is forward primer 5’-ATC CTG TCC AAA CTA AGG CTC G-3’ and reverse primer 5’-ACC TCT TTA GCA TAG TAG TCC GC-3’. Primer sequences specific for Foxp3 is forward primer 5’-CCC AGG AAA GAC AGC AAC CTT-3’ and reverse primer 5’-TTC TCC AAC CAG GCC ACT TG-3’. All primers were designed and synthesized by Invitrogen Corporation. Reaction conditions were 2 minutes at 50°C, 10 minutes at 95°C, followed by 40 cycles of 95°C for 15 seconds and 60°C for 1 minute, finally, 50°C for 15 seconds, 60°C for 15 seconds and 95°C for 10 minutes.

### Flow cytometry

One hundred microlitre heparinized peripheral blood were obtained and red blood cells were lysed to prepare the single cell suspension. All cultured cells from peripheral blood, mediastinal lymph nodes or spleen were harvested, the surface staining antibodies were FITC-anti-CD4, APC-anti-CD25, PE-anti-PD-1 or PECY-7-anti-CTLA-4 and the intracellular staining antibodies were PE-anti-IL-4, APC-anti-IFN-γ, PE-anti-Foxp3 or PECY-7-anti-Foxp3, together with isotype control staining. Meanwhile, after staining, the cells were washed with PBS and fixed in 1% paraformaldehyde to count Th1, Th2 and CD4^+^CD25^+^Foxp3^+^ Treg cells, as well as the expression of PD-1 and CTLA-4 on the surface of Treg cells. All antibodies were purchased from eBioscience Company and procedures were carried out according to the manufacturer’s instructions.

Immunomagnetic beads (Miltenyibiotec, USA) were used to screen out spleenic CD4^+^CD25^+^ T cells from mice in each group, as well as CD11c^+^ cells from mice in the control group, according to the manufacturer’s instructions. CD4^+^CD25^-^ T cells in the control group mice were collected as Teff, which were marked with carboxyfluorescein succinimidyl ester (CFSE; eBioscience, USA). CD11c^+^ cells (2 × 10^4^) and CD4^+^CD25^+^ T cells (1 × 10^5^), as well as CFSE-Teff (1 × 10^5^), were seeded into 96-well round bottom plates (Corning, USA), co-cultured with 1 mg/ml OVA in the incubator at 37°C, with 5% CO_2_ and 95% humidity. After 5 days of cultivation, cells were collected and stained with PE-anti-CD4, rinsed in PBS, and fixed in 1% paraformaldehyde.

All stained cells were detected using the FACScan Flow Analyzer and data were analyzed using FACS express v 3.0 software.

### Statistical analysis

Data were presented as mean ± standard deviation and analyzed using SPSS16.0 software. Statistical comparisons were made by One-Way ANOVA analysis in Figures 2, 3, 4 and 5.

## Abbreviations

SIT: specific immunotherapy; PIT: peptide immunotherapy; MAP: multiple antigen peptide; OVA: ovalbumin; Treg: T regulatory cell; BALF: bronchial alveolar lavage fluid; sIgE: specific immunoglobulin E; sIgG: specific immunoglobulin G; EAE: encephalomyelitis; PBMC: periphery blood mononuclear cell; ICOS: inducible costimulatory molecule; PD-1: programmed death-1; CTLA-4: cytotoxic T lymphocyte associated antigen 4; HE: hematoxylin-eosin; EOS: eosinophils; NEU: neutrophil; LYM: lymphocytes; MAC: macrophages; PMA: phorbol myristoyl acetate; BFA: brefeldin A; FCM: flow cytometry; CFSE: carboxyfluorescein succinimidyl ester; TGF: transforming growth factor; APCs: antigen presenting cells; HRP: horseradish peroxidase; PBS: phosphate buffered saline; TMB: tetramethyl benzidine dihydrochloride.

## Competing interests

The authors declare that they have no competing interests.

## Authors’ contributions

WS and WZ contributed equally to this work. WS conceived of and carried out most of the experiments, WZ conceived of the experiments and performed quantitative PCR, YJZ conducted statistical analyses. All authors discussed the results. ZX designed and directed the project.
